# ‘As soon as you've been there, it makes it personal’: The experience of health‐care staff shadowing patients at the end of life

**DOI:** 10.1111/hex.13107

**Published:** 2020-07-19

**Authors:** Joanna Goodrich, Damien Ridge, Tina Cartwright

**Affiliations:** ^1^ The Point of Care Foundation London UK; ^2^ School of Social Sciences University of Westminster London UK

**Keywords:** end‐of‐life care, patient experience, patient shadowing, patient‐centred care, quality improvement, staff experience

## Abstract

**Background:**

Patient shadowing is an experiential technique intended to enable those who shadow to understand care experience from the patient's point of view. It is used in quality improvement to bring about change that focuses on what is important for patients.

**Aim:**

To explore the acceptability of patient shadowing for health‐care staff, the impact of the experience and subsequent motivations to make improvements.

**Method:**

A qualitative study with a diverse sample of 20 clinical and non‐clinical health‐care staff in different end‐of‐life settings. Data were analysed thematically.

**Results:**

Anticipated anxieties about shadowing did not materialize in participant accounts, although for some it was a deeply emotional experience, intensified by being with patients who were at the end of life. Shadowing not only impacted on participants personally, but also promoted better insights into the experience of patients, thus focusing their improvement efforts. Participants reported that patients and families who were shadowed welcomed additional caring attention.

**Conclusion:**

With the right preparation and support, patient shadowing is a technique that engages and motivates health‐care staff to improve patient‐centred care.

## 1 INTRODUCTION

There has been a drive in recent years to define and improve patient‐centred care^1^, ^2^ with patient experience established as an essential component of quality in health care.^3^ There is a well‐established national patient experience survey programme in the NHS,^4^ but the connection between data collection and improvement has not been strong.^5^ There are a number of reasons why quantitative findings may not have translated into improvements in patient experience, and potential barriers which have been identified include a lack of regular measurement and performance feedback and a lack of experience of interpreting and using survey data.^6^ However, the premise behind the quality improvement (QI) programme which is the focus of this study is that qualitative data are more appropriate for this purpose since there is a need to understand how patients experience a service, in order to improve that experience. The second premise is that experiential approaches to collecting information, such as shadowing, will better enable health‐care staff to understand the immediate experience of care ‘through the patient's eyes’^7^, ^8^, ^9^ and thus make service improvements that will target what is important to patients and their families.

It is known that service improvement approaches introduced to the NHS in recent years have not all been acceptable to staff,^10^ and QI projects can have a negative association with worker satisfaction.^11^ The experience of staff who take part in health‐care QI initiatives is an under‐researched area, including the acceptability of experiential approaches. Researchers in one study observed that evaluations of projects to improve patient experience overlooked a key outcome: ‘deeper, longer term changes in attitudes and behaviours in staff.’^12^ Such changes in staff may also link to changes for patients and might help to understand what engages and motivates health‐care staff to make quality improvements, and how to appeal to their intrinsic motivation, a key area of interest in QI research.^13^ Patient shadowing, a technique involving accompanying patients as they receive care, has been highlighted as potentially having a valuable role in advancing patient‐centred care.^9^ However, health‐care staff perspectives about undertaking shadowing have not yet been explored.

The aims of this study were to:

1. Seek to understand the process of shadowing from the perspective of staff.
2. Explore the experience of shadowing for staff.
3. Explore the impact of shadowing on health‐care staff's knowledge and understanding of the care experience, and their subsequent motivation to make improvements.

### 1.1 Context

The Patient and Family Centred Care (PFCC) programme, first adopted with orthopaedic patients in the USA in 2006,^14^ has been adapted by The Point of Care Foundation, a not‐for‐profit organization that works to improve the experience of patients and staff in the NHS. The participants studied here, members of 19 multidisciplinary health‐care teams from across England, were the fourth cohort to take part since 2010, and the focus of this particular programme was end‐of‐life care, a priority for NHS England (one of the programme's funders) at the time. The programme follows a collaborative learning model, and participants attended three learning events between July 2017 and April 2018. Participants were taught conventional QI methods but in addition a key requirement was for health‐care staff to shadow patients in their service, to inform their understanding of where to focus improvement efforts.^15^ Guidance (both verbal and in a written handbook) was provided beforehand for all shadowers, including procedures to follow if they noted anything of concern.

1 Patients to be shadowed were selected at the discretion of clinical managers locally, and wherever possible, they were unknown to the shadower, although there were occasional exceptions. Consent was gained from them and/or a family visitor.

## 2 METHODS

**FIGURE 1:**
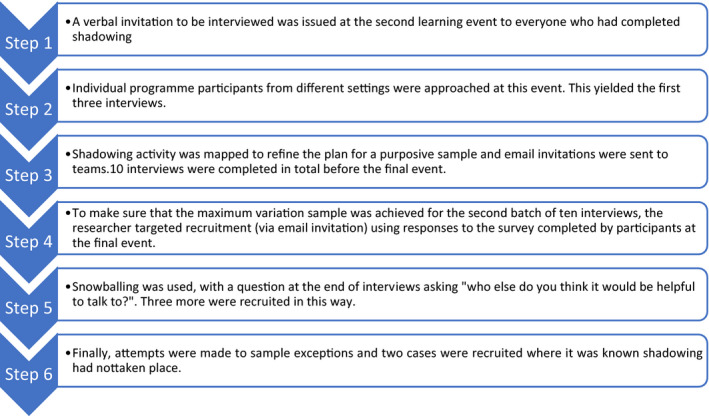
Steps in the recruitment process

Although all nineteen teams who had been selected to take part in the PFCC programme were caring for patients at end of life, they were not working in specialist palliative care units (with the exception of one hospice). The programme was designed for teams working in settings such as hospitals, community and mental health, and nursing homes. The teams were made up of staff with clinical backgrounds (eg geriatrics, general nursing, physiotherapy) and non‐clinical backgrounds, (eg QI and patient experience roles). Most, but not all, had jobs which involved direct patient contact. The programme participants ranged from senior staff who had worked in the NHS for decades, to junior staff who had been working for <10 years. The sampling frame was all programme participants, which was estimated to be a pool of 95 people. The gender balance of the sample reflects the composition of the wider programme. There were two stages of recruitment, early in the programme as shadowing was taking place and later to explore reflections about the process and impact it had on individuals and the service provided. Maximum variation sampling was employed,^16^ with recruitment targeting individuals from a variety of professional backgrounds, length of experience and working across a range of health‐care settings (Table 1: sample characteristics). In response to the written request to be interviewed, one person declined and four did not reply. The final sample of 20 participants was established using a series of steps (Figure 1).

**TABLE 1:** Sample characteristics

Characteristic	Number of participants
Gender	
Female	19
Male	1
Current role	
Clinical (doctor, nurse, AHP)	10
Non‐clinical (eg quality/patient experience)	9
Volunteer/family carer	1
Shadowing setting	
Acute hospital	11
Community/mental health/primary care	3
Hospice	1
Residential/nursing home	3
Other	2
Length of time in health service	
<5 y	2
5‐10 y	3
10‐20 y	4
20‐30 y	6
More than 30 y	5

Three of the participants in the sample had not undertaken shadowing, and the other 17 had carried out shadowing for varying lengths of time, ranging from one session of half an hour to seven sessions of over 1 hour each. They had shadowed at different times of day from early morning to late evening.

A qualitative approach was chosen given the exploratory nature of the study and its focus on the experience of staff undertaking shadowing, which no other study had explored. Data collection was through semi‐structured interviews. These were conducted at the participant's place of work or home, face‐to‐face, by Skype or telephone, according to participant preference. Twelve interviews were by telephone (10 at work, two at home), one by Skype (home) and seven face‐to‐face (at work).

The data from the qualitative interviews^2^ were transcribed verbatim and analysed using thematic analysis (TA),^17^ allowing the researcher to find shared themes across a diverse sample and a broad range of experiences.^3^ Data analysis followed Braun and Clarke's six steps.^18^ The transcripts were coded by hand, line by line. Inductive codes were created, guided by what the participants said, and additional subcodes were created while interpreting the data, drawing on the primary author's professional experience and a review of the relevant literature. These were grouped into themes and subthemes, using an iterative approach involving discussion with the research team, enabling codes to be checked against sample transcripts and further refinement of themes and subthemes. The structure and hierarchy of themes was also shared and discussed with work colleagues responsible for the QI programme which was the focus of the study. This was important for the purposes of rigour but also enabled the researcher, as part of the organization running the programme, to be reflexive and aware of potential issues related to ‘insider status,’ and the need to continuously challenge assumptions.

Ethics approval for the evaluation was obtained from the University of Westminster Ethics Committee.

## 3 RESULTS

Analysis revealed two core themes: the first relates to shadowing as an activity, what was observed by participants, and how shadowers felt before, during and after shadowing. The second relates to the changes brought about by the experience of shadowing, the knowledge and understanding gained, the emotions it evoked and the impact on the shadower (Table 2: themes and subthemes).

**TABLE 2:** Themes and subthemes

Theme →	Subtheme→	Subtheme →	Subtheme
Shadowing as an activity	Observations made	Physical environment Relational care	
	Feelings about doing shadowing	Anxiety	About intruding About seeing poor care About what colleagues would think
		Curiosity ‘give it a go’	
		Doubt/uncertainty	About how to do it About learning anything
	Feelings during shadowing	Positive/enjoyment	
		Uncomfortable	‘Out of role’/personal professional split Being judged by colleagues as ‘slacking off’ Sad situation
	Shadowing style	Intervening Mindful ‘in the zone’ Companion	
Responses to the experience of shadowing	Impact of experience for the project	Increase in knowledge and understanding (cognitive empathy)	
		Increase in affective empathy	
	Personal impact	Motivation	To make improvements for patients Re‐engagement with own work
		Thoughts about own dying, death and mortality	
	Subjectivity of observation	‘Lens’ affecting interpretation	Personal experience Professional experience Personality
		Emotional response affecting interpretation	

### 3.1 Shadowing as an activity

#### 3.1.1 What they saw: observations made during shadowing

Observations made by participants about the environment of care fell broadly into two categories: the physical environment and the relational care, or how they saw people connecting with each other. Participants noticed aspects of the environment which they had not previously been aware of as staff: how it looked, sounded, smelled or felt. Several mentioned the lack of visual stimulation for patients and families, while at the same time being struck by ‘hospital clutter.’ Other participants noticed details about the patients' immediate environment, such as difficulties with reaching call bells (which was noted as an easy issue to fix), noise levels (eg associated with hospital bins), music played in the background and especially the lack of privacy.

[The doctor] is having an end of life discussion with someone and so everyone in that bay knows [that person] is going to die. [female, doctor]

A recurring theme specifically related to how appropriate the environment looked or felt for patients who were dying, whether in bays, onwards, hospital side rooms or nursing home single rooms. In a nursing home room, the environment was described as dreary: ‘the room was kind of sparse.’ While on the ward, the conditions were notably cramped:

For the family members, you are in a confined space and you have these nasty blue curtains around, and you can hear everything else going on, you know, it's not quite what you need. [female, OT assistant]

Relationally, shadowers noticed how the way staff communicated with patients and their families varied, with mixed results. Many shadowers noticed how there was little contact between patients and staff, and often used the word ‘lonely’ to describe how they imagined the patient might feel. The word ‘lonely’ appeared to encompass emotional and physical dimensions. There was the sense that a patient felt alone (which might have been more keenly felt because a patient was dying) and was not receiving comforting words or touch. There was also a sense of patient isolation in relation to surrounding activity. It was reported that single rooms in hospitals or residential homes could appear physically cut‐off and that patients sometimes might have felt forgotten. However, these observations were also made about patients' experience in bays on wards despite the surrounding physical activity:

a hubbub of stuff going on.…you probably get no emotional stimulation…literally, you know the nicest thing is when someone came and held a hand, or just straightened the covers, you know, just that touch, that human contact. [female, doctor]

Boredom was another aspect of isolation and loneliness and came as a revelation for some shadowers:

I feel busy on the wards when I'm there as a doctor, you know, crazy busy. From a patient's point of view, you're lucky if you see a nurse, let alone be able to talk to a nurse or communicate to a nurse. It's really lonely. [female, doctor]

Shadowers noticed how there were long periods for the patient where nothing apparently happened. In a side room, a shadower realized how a patient spent hours looking out of the window:

looking at the shape of the clouds and how they moved…because their day is so long isn't it? [female, patient experience role]

However, in a hospital ward environment, a participant noted how attentive some patients were to interactions between staff members:

They notice the conversations between staff, and the facial expressions, they see all of it, the raised eyebrows, everything. [female, patient experience role]

Participants noticed that routine processes or tasks could be enhanced for patients with positive accompanying interactions and demonstrations of kindness.

I watched a healthcare assistant come in and ask if he wanted a drink… she was very caring, she had a little chat with him about would he like to sit out later on, because she knew he was up earlier that morning and went back to bed. [female, nurse]

Food, and the way it was provided via routines, was a particularly strong theme. The moments of contact when food or a drink was provided could play an important part in the patient's day, and for patients at end of life who are not able to eat much solid food, or eat at all, it took on particular significance if not provided sensitively.

#### 3.1.2 How participants felt about shadowing

Most shadowers had intellectually understood the purpose of shadowing, yet attitudes before commencement varied from sceptical to curious, to positive. Some reported feeling privileged to be able to have the opportunity to shadow and described how they embraced the chance to see an aspect of service delivery ‘through a different lens, not just a healthcare professional lens.’ Others were willing to ‘give it a go’ or volunteered to shadow in the hope it would make a difference to patients' experience. One specifically volunteered because she saw shadowing as providing companionship for dying patients:

I hate to see or think of people being on their own and having no‐one. So, although I would be shadowing, I might well be holding someone's hand at the same time…I think it might have eased them, given them some comfort. [female, hospital volunteer]

Interestingly, in spite of the willingness to try shadowing, the most often cited emotions before shadowing were apprehension, anxiety and worry. The most common concern was about being intrusive or unwelcome:

I didn't want to put anybody in a difficult position and I didn't want to be put in a difficult position. [female, quality improvement role]

Shadowing patients who were dying intensified these concerns, particularly anxiety expressed by clinicians and non‐clinicians alike about whether it was appropriate to intrude at such a private time, when there may have been only a few days left for the patient and their family. This appeared to reflect a concern about the patients and about the shadower's own assumed awkwardness in the situation and was the predominant reason given by those who did not shadow for their decision. Secondly, others were worried about upsetting colleagues, who might feel that they were being observed and judged, ‘to make sure they do things in the right way.’ Thirdly, fear of seeing poor care was an issue. Here, there was apprehension that it would upset their own equilibrium if they witnessed and would have to report poor care in their service (which in fact did not happen^4^):

I was a little bit concerned at first because I kind of knew I wouldn't like what I was going to see. [female, OT assistant]

Finally, a minority of participants were unsure how the process of shadowing would function. For example, there were those who described themselves as ‘doers’ and felt they would not have the patience to watch. Another reported being a ‘shy person’ and was unsure how she would be able to interact with the patients and families while shadowing.

For the majority, initial anxieties and fears proved to be unfounded. For example, the participant who had thought it would be difficult to ‘do nothing’ found the time went more quickly than expected. Several spoke of finding the experience less awkward or intrusive than they had feared and reported that patients and families who had agreed to be shadowed welcomed them. A non‐clinician spoke of the experience as ‘lovely’ and rewarding, because of the quality of the relational care she observed, which she felt was kind and compassionate. Others spoke of enjoyment and that the experience of shadowing was a privilege. There was only one exception where a participant spoke afterwards about feeling awkward:

You know, the nature of the care is quite intimate and you know it's uncomfortable. You witness things that perhaps you feel you shouldn't witness. [female, quality improvement role]

Support mechanisms were put in place by project leaders in case shadowers found the experience distressing, but support was not taken up beyond the shadowing team's debrief. Nevertheless, feelings evoked were not always easy, with one participant saying ‘it was quite sad and upsetting at times,’ with another describing a conversation she witnessed about advance care planning as ‘…very moving to watch..it was difficult for me yes, although I managed to keep myself in check.’ [female, commissioner]

Although some, particularly senior clinicians, were aware that their colleagues might feel that they were being watched, conversely some shadowers spoke of feeling self‐conscious about how their colleagues perceived what they were doing. There was concern that colleagues might have a negative attitude towards shadowing, as it might be mistaken for ‘slacking off.’ For those who reflected more deeply on the experience, it was stepping out of the professional role that appeared to be a key reason for discomfort. One of the study participants, a doctor with over 40 years of experience, reflected that it felt ‘just a little bit unusual.’ He went on to reflect further, suggesting he was in a situation which was not the usual relationship with a patient, and which involved stepping out of his professional role, which he found ‘interesting but not difficult.’ Another participant—a nurse—spoke of the challenge of taking off the professional ‘hat’ when shadowing.

### 3.2 How the experience of shadowing brought about change for patients and staff

Shadowing appeared to engender empathy, that is an assumed capacity to understand or feel what another person is experiencing, or to place oneself in another's position. Both cognitive empathy (how we understand other people) and affective empathy (our emotional reactions to people^19^ ^)^ were claimed by participants to increase understanding and emotional engagement with patients, as outlined below:

#### 3.2.1 Work as imagined and work as done: cognitive empathy

When reflecting on the experience of being with patients, participants talked about it increasing their knowledge or understanding of what patients and families went through. Some spoke of their surprise to discover it differed from what they had expected. A striking example was how busy clinical staff became aware that for patients ‘nothing happened’ for long periods of time. Other practical examples were call bells being out of reach, inappropriately large meals being delivered and taken away again uneaten, or conversations at the nursing station being audible. This kind of increased understanding, or tacit knowledge, was a sign of increased cognitive empathy.

Participants spoke about the benefit of taking time to step out of their normally busy day, and how this enabled previous modes of operation to be challenged: ‘we get caught up in just the doing, without stopping and thinking about what happens to people’ [female, nurse]. This new tacit knowledge translated into a commitment to making practical changes, for example, one ward changed its practice of automatically putting dying patients in side rooms, and another team changed the way food was offered. There were some reflections from clinicians that it was particularly valuable to be challenged by the ‘fresh eyes’ of non‐clinical colleagues. Shadowing made an impact on participants professionally as well as personally, and changes for patients and families were made as a result.^5^ For example, an OT assistant who undertook several hours of shadowing (six or seven patients) described how it had changed her behaviour, so that even if she was not sitting and shadowing she would now ‘always be watching things and looking out.’ She felt she could recognize signs that a patient was nearing the end of life, and felt more confident about informing the doctors.

#### 3.2.2 Emotional response: affective empathy

Although none of the participants used the word empathy, affective empathy—even if not described in this language—was seen in many participant accounts. Here, some responded with sadness or frustration, indignation or even anger on behalf of patients. It was demonstrated too when they described how they connected with particular patients or relatives they shadowed. For instance, one participant revealed how she became involved in the struggle of the patient, ‘She looked very vulnerable and basically you wanted to scoop her up and take her away’ [female, nurse]. Another participant spoke about an experience of shadowing being upsetting, because she identified with the family of the patient she was shadowing as like her own family. Others spoke about how the patient, or a moment, had ‘stayed with them’ in a way that is different from meeting patients under other circumstances: ‘It goes into a part of your brain that you remember what you've seen.’ [female, health‐care assistant]

#### 3.2.3 ‘If I was in that bed’: subjectivity of observations and empathy

Although most shadowers consciously tried to imagine what it was like to be the patient (to ‘get in the zone’), each individual brought something of their own to shadowing in terms of professional role or training, personal experience, personality or outlook, that is the ‘lens’ through which people perceived or interpreted what they were seeing. Some brought personal experience of bereavement, death and dying, and expressed their experiences in the light of this:

I do remember being with my grandma when she died in hospital….I went home [after shadowing] and talked about it to my husband and reflected that actually it had given me a different perspective on dying in a hospital. [female, quality improvement role]

One participant was aware of the risk of identifying with patients and commented that ‘we are not that person’ [female, patient experience role]. Most others, however, were not aware of such risks, and sometimes began comments with phrases like ‘if I was in that bed…,’ going on to make potentially inaccurate judgements. Sometimes, identification could be used positively. An OT assistant said that she tried to treat others as she would like herself or her family to be treated, and when shadowing viewed what she saw while thinking ‘if it was my mum…’ Differing views about whether it was better for dying patients to be in a side room on their own, with closed blinds and dim lighting, or on a busy ward with music playing illustrates how varied personal preferences could be.

#### 3.2.4 Motivation

One manager noticed how her team engaged emotionally with shadowing and that it had given her staff ‘a thirst for quality improvements, to look at changes for improvements that they can engage in and make to improve patient experience’ [female, nurse]. A key characteristic of this QI approach was that the project teams met together after shadowing. This enabled them to identify where change could be made, suggest ideas for improvement and then make the changes: ‘Well as soon as you've been there, it makes it personal. So then all of a sudden you're wanting to do something’ [female, doctor]. Others reflected that it was rewarding to be able to make a tangible contribution to care and that they felt a renewed motivation for work immediately after shadowing:

It made a connection with why you're doing it [being a doctor] in the first place. I think it took the professional side of it away and brought the emotional side in. [female, doctor]

## 4 DISCUSSION

This study has elaborated on the processes, key experiences and impacts of shadowing, and has revealed the significant place of emotion in this work. Our paper uncovered the nature of the most challenging aspects of the work of shadowing for health‐care staff, which proved to be emotional, rather than practical, professional, logistical or ethical. The emotions felt by shadowers in response to their experience of being with patients in this way were complex, and at the heart was the way that new perspectives afforded were ‘unusual.’ Participants found they were accompanying patients, seeing the familiar from unfamiliar vantage points, which created new emotional responses to the patients and what they were experiencing. They appeared at times to be taking on informally a role which was different from that of observer. The physical and emotional closeness that comes with shadowing seemed to open up varying relational spaces and opportunities for an emergent meaning‐making and knowledge that would not have been available in other ways. For example, the satisfaction expressed through providing companionship to a patient who was lonely or bored. Such ‘relational goods’ are not things, ideas or services, they are emergent and have a ‘sui generis reality’ (ie an unique dynamic): they are productive, have benefits and cannot be appropriated by any one single individual.^20^ Emotional investments that arise in relational moments could subsequently play a part in motivating participants to make changes for their patients for the better, including engaging in their projects in a way not usually seen in QI, which if managed appropriately could be effective for service improvement. A team leader, reflecting on shadowing as an activity, said of her colleagues: ‘It gives them a thirst for quality improvements, that they can engage in and make to improve patient experience.’ [female, nurse]

### 4.1 The place of emotion

Beyond the practical aspects of shadowing, this study points to deeper fears that underpin the practice, of being in unusual and uncomfortable situations with patients, where professional identity becomes irrelevant, and so cannot provide insurance against awkward situations. Even though ‘the development of necessary professional detachment’^21^ is no longer taught formally to medical and nursing undergraduates, detachment is still acknowledged as part of the ‘hidden curriculum’^22^ and as a mechanism for coping with the nature of the work health‐care staff do, and there is debate about how too much emotional involvement with patients can lead to burnout.^23^, ^24^ In studies of trainee doctors, for example, it has been noted that detachment increases over time.^25^ Shadowing represents a challenge to this detachment. Menzies‐Lyth^21^ went further, describing how defences are a natural reaction to the anxiety of caring for, and being in constant close proximity to patients who are sick, suffering or dying, and this was suggested in the comment of one participant: ‘there is a fear of empathizing too much.’ Participants who intervened or made judgements from a clinician's point of view were not shedding the protection of their professional role. Indeed, to a greater or lesser extent, some participants found ways (whether intentional or not) of resisting the possibility of shadowing breaking through their professional detachment. One of these may have been ‘comfort seeking,’^13^ when shadowing became merely an exercise in reassuring themselves that care is good, or not as bad as they had feared. However, many clinical participants did respond emotionally, and it would be interesting to explore further how far professional training, personality and life experiences contributed variously to their response.

The importance of reflexivity for researchers when shadowing has been highlighted,^9^ and this study points to how it would be equally valuable for non‐researchers who undertake shadowing to reflect before, during and after shadowing, on how they are bringing aspects of themselves to what they observe. Reflexivity, ‘critical assessment of presuppositions,’ is not consistently taught as part of clinical training,^26^ and in any case, the concept of reflexivity in relation to shadowing could be adapted to be built into training, preparation and debriefing for participants. Likewise, the emotional response demonstrated by shadowers, while being a positive element for change because it appears to galvanize health‐care staff into action, should, however, be treated cautiously. The context for the patient and the patient's preferences may not be the same as the shadower's. It has been said that it is never possible to see through the patient's eyes, as one of the participants said, ‘you are not that person.’ It is not possible to know completely the patient's context, their life experiences until this point, or for the (usually) younger and healthier person who is shadowing, how it feels to be very ill or dying. A lack of reflexivity could mean in some cases mistaking over‐identification for empathy.

### 4.2 Motivation

This study has illustrated the relationship between emotional response, increased understanding and motivation. A review of formal evaluations of programmes to improve quality in health care identified factors needed for success and identified 10 challenges; the first of which was convincing clinical teams that there is a real problem to be addressed.^27^ It was recommended that ‘ those designing and planning interventions should be careful to target problems that are likely to be accepted as real’ and suggested using patient stories to secure emotional engagement and engage the clinicians in defining what they would like to improve. Shadowing appears to achieve these goals.

We found that the experience of shadowing and being with patients at end of life has a strong emotional impact for some staff, which increases their motivation to engage with the improvement programme, and to make the experience of patients and their families better. The concept of intrinsic motivation may illuminate the link between empathy and motivation to engage in improvement efforts. Herzer and Pronovost^28^ have asserted that QI initiatives, if they are to engage doctors/clinicians, must ‘light the intrinsic fire.’ Shadowing appears to lead to empathy—or at least identification—and sparks a desire to consciously provide kinder and more compassionate care for patients, and to make changes to achieve this. Participants spoke of being reminded through shadowing of why they had wanted to work in health care. Participants in the study found it rewarding to be able to understand how the experience of patients could be improved and then to make the changes themselves. Often data are provided about patient experience, or staff are asked to make improvements, but the emotional engagement is lacking. This study makes a case for introducing shadowing to the range of tools available for collecting data, and co‐production of better services to improve patient experience, by emphasizing ‘the person in the patient.’^2^

### 4.3 Limitations and strengths

The study did not aim to discover the experience of those being shadowed, so patients and family members were not interviewed; this could be a focus for further research. However, participants noted that patients at the end of life, and their families, generally appeared to appreciate being shadowed as they felt it was a demonstration of attention to their experience of care. The response rate was high overall, and the sample represented the gender distribution of the programme, but further research might explore a programme with more male participants. Time pressures on busy health‐care staff were apparent, and although efforts were made to conduct as many face‐to‐face interviews as possible, away from the immediate work environment, it was a pragmatic decision to conduct telephone interviews at times that suited the participants. Nevertheless, in spite of the practical difficulties, the interviews yielded a valuable depth of data, and this is the first study to explore the accounts of health‐care staff shadowing patients.

### 4.4 Implications of findings for practice

This study demonstrates the importance of preparing staff for the emotional impact of shadowing, in conjunction with the current practical guidance. Although this study's context is specific to patients at the end of life (and therefore brings specific challenges), it has highlighted that shadowing, probably in any context, is an activity that places staff in a situation with their patients that is different from usual and that it might be uncomfortable to step out of role in this way. Discussion should cover this challenge to professional detachment.

The anxieties that staff reported before they began shadowing can be addressed. Fear of being intrusive, fear of what their colleagues might feel about being shadowed (and self‐consciousness about shadowing not being ‘proper work’) can be addressed through preparing patients and families beforehand, and by approaching them sensitively to ask permission while explaining that they can decline to be shadowed. Colleagues can be prepared by explaining the purpose of shadowing. Fear of seeing poor care can be discussed beforehand, and a process agreed should this happen. Guidance should be given on the importance of providing emotional as well as practical support for those who might find shadowing difficult, or personally challenging, both before and after shadowing. Team leaders need to support the shadowing process and advise on how to respond to the emotional impact.

Additionally, there should be guidance on how personal factors might influence judgement (reflexivity). Ways of mitigating this would be to encourage shadowing in pairs, standardize logging observations and reframing shadowing as a group activity. Emphasis can be placed on the importance of debriefing, and sharing and discussing the implications of what has be learned for changes in patient care. Members of the project team will each bring valuable observations which could be interpreted in different ways, thus offering a more holistic perspective. For example, if one team member responds with cognitive empathy (observing faults in processes or broken equipment) and another with affective empathy (noticing how patients did not receive comforting touch or words), this could be combined to create a deeper understanding of patients' experience, and how to improve it. It appears that, in terms of wanting to make changes to the patient's experience, it did not matter whether cognitive or affective empathy was elicited. Shadowing should be a team activity for this reason. Team debriefs are also an important source of support in case the experience of shadowing has been difficult.

## 5 CONCLUSION

Initial anxieties and fears about shadowing appeared to be generally unrealized, and many spoke of it being a rewarding experience, and that it ‘reconnected’ them with patients and their own motivation to care. Shadowing appears to be an acceptable approach to QI which engages staff. For some it had a powerful personal impact emotionally, intensified for some by shadowing patients who were dying. Participants reported increased understanding of the experience of care and went on to describe improvements they had made to the care experience for patients and families. Shadowing enables cognitive and affective empathy with patients, which combined, works to motivate staff, who with the right preparation and support, can develop the skill to make real changes to patients' and families' experience of care.

## Data Availability

The data that support the findings of this study are available on request from the corresponding author. The data are not publicly available due to privacy or ethical restrictions.

## Footnotes

^1^ It was made clear that shadowing did not prevent them from caring for the patient if needed.

^2^ The data that support the findings of this study are available on request from the corresponding author. The data are not publicly available due to privacy or ethical restrictions.

^3^ There was no PPIE in the design or conduct of the study.

^4^ The data included two examples of participants who followed the agreed procedure and spoke to the ward manager about concerns about poor behaviour of staff (rather than poor care).

^5^ This study was not an evaluation of changes made but examples are included in the Appendix 1.

## Appendix 1

### Examples of changes recorded by teams in this study

#### Hospital team 1

- Increased use of priorities of care individualized care plans for patients in last days of life.
- Improved development of individualized advanced care plans for patients discharged home.
- Lockdown lunch where all staff, including managers, come to the ward to help patients eat.
- Open visiting times to enable visitors to care for loved ones.
- Increased drive to recruit more volunteers to help with emotional support and activities, and ‘adopt a grandparent’ scheme.
- Safety huddle introduced to improve communication within MDT regarding challenges of that day.

#### Hospital team 2

- Musicians invited to wards to improve the boredom and environment.
- Work to improve information board on wards to improve communication between MDT.
- Employed a new role on the wards to reduce complex discharge delays.
- Use of blue plates as proven to encourage eating and thus improve nutrition.

#### Hospital team 3

- Purple Butterfly sign for room door to highlight to staff that a patient was nearing end of life.
- Purple Butterfly symbol on electronic flow board to highlight to bed managers and other professionals that there were dying patients on the ward.
- Purple Butterfly sticker on drug charts to those pharmacists prompted to check ward drug stocks and charts processed quickly through pharmacy.
- “Tell us about you” form to encourage staff to ask patients about personal things that are important to them in advance care planning.
- New symptom chart so that patients are asked a number of patient‐centred questions.
- Delivery of ward‐based training for staff and half‐day communication skills workshops to improve confidence in having conversations about end of life care.
- Development of ‘after death huddle’ at each shift handover to talk about what happened, what went well and what could have been improved.

#### Hospital team 4

- Call bells in reach.
- Small meals offered.
- New beds for family to stay.
- Bedsides decluttered.
- Designated parking for relatives visiting patients near end of life.
- Provision of food for relatives and a quiet room.

#### CCG

- ‘Red bag’ initiative for care homes, with all necessary information and paperwork for advance care planning, with training rolled out across CCG.
- General communication training being rolled out across CCG.
- Care home bedrooms refurbished.
